# Deaths from Chloroform

**Published:** 1852-08

**Authors:** 


*Deaths from Chloroform.*—In the *Boston Medical and Surgical Journal*, May 19th, Dr. A. E. Noble reports the following case:

In this instance he [the operator] gave a drachm on a handkerchief, which the patient inhaled without any apparent effect, and he then added about half a drachm, I watching the effect closely. After inhaling again a few times, she said she felt the effect a *little*, but the operator and myself could not perceive any influence. Yet willing she should think that she would not experience any pain, the instrument was applied, and as the tooth was extracted she raised her hands to her face and exclaimed "Oh!"

then letting her hands drop, she sat erect about six seconds, when her head dropped upon her breast. I felt for her pulse, but there was none. She respired not more than three or four times, and was dead! It did not exceed three minutes from the first inhalation until life was extinct. That the effect of chloroform was progressive in this case, is certain. I could detail many circumstances relating to the case, such as age, complexion, apoplectic predisposition, and other physical matters, but do not conceive it to be of interest in these days of chloroform. I will add that she was a resident of Detroit, but was in this place on a visit.

A fatal case in obstetrical practice is reported to have occurred recently in Georgia, from the same cause. And at Stamford, Conn., Mrs. Wood, wishing to have a tooth extracted, requested the dentist to administer chloroform. He complied with her wishes; after inhaling it a few times she sunk back upon the sofa and expired.

